# The Behavior of Some Bacterial Strains Isolated from Fallow Deer Compared to Antimicrobial Substances in Western Romania

**DOI:** 10.3390/antibiotics12040743

**Published:** 2023-04-12

**Authors:** Emil Tîrziu, Alexandrina V. Bulucea, Kalman Imre, Ileana Nichita, Florin Muselin, Eugenia Dumitrescu, Andreea Tîrziu, Narcisa G. Mederle, Alexandru Moza, Iulia M. Bucur, Romeo T. Cristina

**Affiliations:** 1 Faculty of Veterinary Medicine, Calea Aradului 119, 300645 Timisoara, Romania; 2 Faculty of Medicine, University of Medicine and Farmacy “Victor Babes”, Piata Eftimie Murgu No. 2, 300041 Timisoara, Romania

**Keywords:** sylvatic, zoonotic antibioresistance, monitoring, one-health, *Dama dama*, *E. coli*, Vitek-2 Compact

## Abstract

(1) Background: The resistance levels of *Escherichia coli*, *Salmonella* spp., *Pseudomonas* spp., *Staphylococcus* spp., etc., isolated from the nasal cavity and the rectum of *Dama dama* deer from three hunting grounds in Western Romania were assessed. (2) Methods: The analysis was completed using the diffusimetric method, compliant with CLSI reference standards, and with Vitek-2 (BioMérieux, France), on 240 samples. (3) Results: The results were statistically analyzed (by one-way ANOVA) revealing that in four of the ten *E. coli* strains isolated from animals, 87.5% (*p* < 0.001) resistance was found. *E. coli* strains were resistant to cephalexin (100%); seven strains were resistant to cephalothin and ampicillin; six were resistant to cefquinome and cefoperazone; five were resistant to amoxicillin/clavulanic acid; and four were resistant to ceftiofur. However, *E. coli* was sensitive to amikacin (100%). The most efficient structures were beta-lactams, amikacin, and imipenem, to which all 47 strains studied (100%) were sensitive, followed by nitrofurantoin, to which 45 strains (95.7%) were sensitive, neomycin, to which 44 strains (93.6%) were sensitive, ceftiofur, to which 43 strains (91.5%) were sensitive, and trimethoprim/sulfamethoxazole and marbofloxacin, to which 42 strains (89.4%) were sensitive. (4) Conclusions: In wild animal populations, where a human presence is frequently reported, including a constant presence of domestic animals, despite the perceived low risk of emerging resistance to antimicrobials, resistance is likely to develop frequently.

## 1. Introduction

Antibiotic resistance is reported frequently, in numerous species of bacteria, and represents a very topical problem, both in veterinary medicine and in human medicine, being considered a worldwide issue, with an extremely marked zoonotic risk [1,2,3]. Lately, the resistance phenotypes of animal pathogenic bacteria have continuously increased due to the indiscriminate use of antibiotic-based veterinary medicinal products, especially in intensively raised animals [4,5,6].

Antibiotics represented and still represent the most effective means of combating pathogenic bacteria. However, since the beginning of their use, cases of antimicrobial resistance have been reported, and the phenomenon has become downright alarming in recent decades [7]. Thus, currently, due to the fact that bacterial resistance is a constantly growing problem, obtaining new data, regarding combating microbial resistance, has become a “necessity” for both human and veterinary medicine [8].

Resistance to antibiotics can be classified in two categories and is influenced by several factors. From the moment of exposure to an antimicrobialagent, bacteria develop resistance mechanisms, and this biological natural process leads to the survival of the most resistant strains [9,10].

Numerous types of research, carried out in recent years, demonstrate that the phenotypic and genotypic resistance, present in many species of bacteria, isolated from domestic animals and humans, is also found in a significant proportion in some isolated from wild animals, including those raised in a farm system. Although antimicrobial resistance, considered one of the most important global threats facing humans and domestic animals, is well studied, the role of wildlife in its transmission requires further study [11,12].

Though it was suggested that wild animals are not exposed to direct selection pressure, through the therapeutic use of antimicrobials, it has been proven that, in their intestinal flora, germs resistant to the action of antibiotics can be found. This finding suggests that resistance to antimicrobial agents has spread beyond human and domestic animal habitats [13]. For this reason, understanding the influence of wildlife, regarding the epidemiology of resistance to antimicrobial substances, can elucidate some of the mechanisms of emergence and transmission of bacteria resistant to antimicrobial agents, which significantly have a particular impact on human and domestic animals’ health [13].

Although an emerging global problem, antimicrobial resistance in wild animals is rarely seen due to their seldom exposure to antimicrobials. However, the increasing interactions of these animals with humans and domestic animals can have a huge impact on bacterial flora [14]. For example, in an eight-year study in Northwest Italy, whose main aim was to investigate the presence of *Salmonella* spp. isolates in wild animals, including their antimicrobial resistance, it was found that of the 88 strains tested, almost all (97.7%) showed resistance or an intermediate resistance to at least one class of antibiotics [15].

The componds were selected according to their relevance to public health and taking into account the recommendations of EFSA [16], the Enter-net Italia network [17], and data available in the specialized literature. Finally, the authors mention that among the antibiotics tested, the highest resistance values were recorded for antimicrobials from the tetracycline class [15]. Additionally, commensal bacteria act as important sources of virulence and resistance genes. However, existing data are generally focused only on the analysis of human bacteria or related to human communities. There are, most researchers argue, relatively few genomic studies of commensal bacteria from hosts less exposed to antibiotics, such as wildlife [18].

Considering the aspects mentioned above, research on multiple resistance to antimicrobials, of bacterial strains isolated from fallow deer populations, is of particular importance, simultaneously with the epidemiological monitoring of strains isolated and identified from males and females of different ages.

In this context, the aim of the research that was carried out was to evaluate the levels of resistance to antibiotics of some strains of *Escherichia coli*, *Salmonella* spp., *Pseudomonas* spp., *Staphylococcus* spp., etc., isolated from the nasal cavity and from the rectum of stags and fawns, belonging to the species *Dama dama*, of different ages, immediately after shooting, from three hunting grounds in the west of the country. The evaluation and interpretation of the results were done by the diffusimetric method, in compliance with the CLSI reference standards and with the help of the Vitek-2 system (BioMérieux, Craponne, France) [10,19].

## 2. Results

### 2.1. The Antimicrobial Susceptibility after the Diffusimetric Method

#### 2.1.1. Socodor Hunting Ground

In order to test the susceptibility of Gram-negative species, isolated from samples collected from the Socodor hunting ground, to antimicrobials, five strains of *Escherichia coli* were studied. These included three strains in the genus *Enterobacter*, one strain of *Pseudomonas oleovorans*, and one of *Providencia rettgeri*. From the other two hunting grounds (Chișineu Criș–Sălișteanca and Nadăș), five strains of *Escherichia coli*, from each area, three of *Salmonella* spp. and two of *Enterobacter* spp., were tested.

Compared to the isolates from the hunting grounds Chișineu Criș–Sălișteanca and Nadăș, in the strains of *E. coli* isolated in the animals from the Socodor hunting grounds, significantly higher resistance to most of the antibiotics tested was found. This was between 50 and 90%. Thus, as can be seen from Table 1, all five *E. coli* strains studied were 100% resistant to ampicillin, amoxicillin/clavulanic acid, ciprofloxacin, and azithromycin. The only molecule to which all *E. coli* strains were sensitive was ceftazidime (100%). The bacteria were sensitive to the other compounds in different proportions: three strains (60%) were sensitive to nitrofurantoin; two (40%) were sensitive to trimethoprim/sulfamethoxazole and chloramphenicol; and only one strain (20%) was sensitive to cefuroxime.

**Figure 1:**
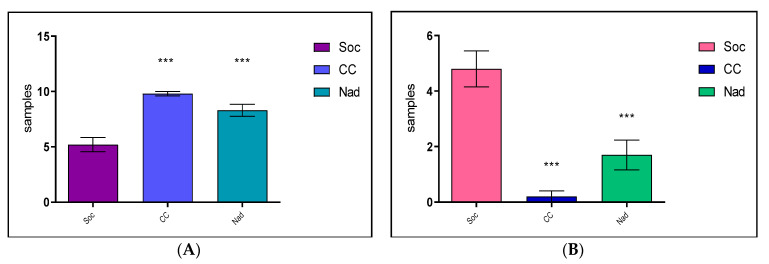
Comparative Sensitivity (**A**)/Resistance (**B**), in studied hunting grounds after the difusimetric method (Where: *** means *p* < 0.001, Soc = Socodor, CC = Chișineu–Criș, Nad = Nadăș hunting grounds).

The statistical analysis of sensitivity vs. resistance in the case of the Kirby–Bauer disk diffusimetric method confirmed the presence of resistance in the Socodor hunting ground (*** *p* < 0.001), compared with the other two studied areas (Figure 1).

The other species, among the Gram-negative bacteria isolated from deer in the Socodor hunting grounds, showed insignificant resistance. Thus, *Pseudomonas oleovorans* was sensitive to all ten antimicrobial substances tested (100%); *Enterobacter* spp. was sensitive to nine out of ten substances tested (90%), resistance being found only to ciprofloxacin (10%); and *Providencia rettgeri* was resistant to two antimicrobial agents (20%), namely cefuroxime and chloramphenicol, and sensitive to the other eight substances (80%).

#### 2.1.2. Chișineu Criș–Sălișteanca Hunting Ground

In this case, the experiments performed on the samples isolated from Chișinău Criș–Sălișteanca hunting ground showed, with the exception of two strains of *E. coli*, that all species isolates were sensitive to the antimicrobial compounds tested, the prevalence being between 90 and 100% (Table 1).

These results lead to the conclusion that in the animals from the Chișinău Criș–Sălișteanca hunting ground, during the experimental period, only wild strains circulated, an aspect also confirmed by the absence of infectious diseases in animals from this area.

#### 2.1.3. Nadăș Hunting Ground

Results similar to those recorded when testing the bacterial species isolated in the samples from Chișinău Criș–Sălișteanca were also found in the Nadăș hunting ground. Antibiotic resistance was present in only eight of the ten strains studied, with the mention that the resistance to the antibioticstested was moderate, between 10 and 30% (*p* < 0.01).

Of the ten strains tested, two (*Escherichia coli* and *Salmonella* spp.) were sensitive to all ten antimicrobials tested. Two other strains of *E. coli* were resistant to three antibiotics each (ampicillin, cefuroxime, ceftazidime, and trimethoprim/sulfamethoxazole), and one was resistnat to two antibiotics (ampicillin and gentamicin). An aspect also observed was that two of the three strains of *Salmonella* spp. were resistant to ampicillin, gentamicin, and azithromycin, and two of *Enterobacter* spp. were resistant to cefuroxime and azithromycin. It can be said that antibiotic resistance was present in the strains of *Escherichia coli*, isolated from Socodor, including the three strains belonging to the genus *Enterobacter*. Thus, compared to the isolates from the hunting grounds, Chișinău Criș and Nadăș, the *Escherichia coli* strains isolated from the Socodor showed significant resistance to most of the antimicrobial compounds tested, among 50 and 90% (*p* < 0.001) (Table 1).

### 2.2. The Antimicrobial Susceptibility of Some Gram-Negative Bacterial Species with the Vitek-2 Compact

The results of the research on the antimicrobial susceptibility of Gram-negative bacterial strains, taken from deer and fawns from the three hunting grounds, were classified into three categories of susceptibility: sensitive, resistant, and strains with intermediate resistance (after the EUCAST guidelines) [9]. After carrying out the experiments, the results obtained following the testing of 47 strains, isolated from the nasal and rectal cavity, highlighted a very variable susceptibility from one hunting ground to another and from one bacterial species to another.

#### 2.2.1. Socodor Hunting Ground

The 15 Gram-negative bacterial strains, more frequently isolated in the Socodor hunting ground, were classified into four genera: *Escherichia*, *Enterobacter*, *Providencia*, and *Pseudomonas*. Of the 15 strains, ten belonged to the species *Escherichia coli*, in which significant resistance to most antimicrobial molecules was found (Table 2).

To study the behavior of the *Escherichia coli* species toward beta-lactams, the following markers were used: aminopenicillins (amoxicillin, ampicillin), aminopenicillins with beta-lactamase inhibitors (amoxicillin + clavulanic acid), cephalosporins of the first generation (cephalexin, cephalothin), third generation (cefoperazone, ceftiofur), and fourth generation (cefquinome), and carbapenems (imipenem).

Thus, in four of the ten strains of *Escherichia coli*, a resistance of 87.5% was found to seven beta-lactams (ampicillin, amoxicillin/clavulanic acid, cephalexin, cephalothin, cefquinome, cefoperazone, ceftiofur). The only molecule to which all strains were sensitive was imipenem. Among the eight beta-lactams, all *Escherichia coli* strains studied were resistant to cephalexin (100%). Seven strains (70%) were resistant to cephalothin; the other three developed moderate resistance, with no susceptible strains.

With aminoglycosides, *E. coli* strains also behaved differently. Thus, all were sensitive to amikacin (100%), and seven (70%) were sensitive to neomycin. Conversely, to gentamicin and flumequine, five were sensitive, and were five resistant (50%); also, among the resistant ones, there were those that developed significant resistance to beta-lactams as well (Table 2).

For quinolones, the results were similar to those found for aminoglycosides; namely, five strains were sensitive to the two substances, and five strains were resistant (50%). Resistance was recorded in the same strains that were resistant to both beta-lactams and aminoglycosides. These strains were also resistant to tetracycline and trimethoprim/sulfamethoxazole (Table 2).

Trimethoprim/sulfamethoxazole, whose clinical efficiency is not fully proven but recommended by most therapeutic guidelines, can be a therapeutic option in the case of multiresistant *Escherichia coli* strains, taking into account the high sensitivity to in vitro testing.

It can be remarked that, in the strains of *Escherichia coli* isolated from deer in the Socodor hunting ground, there were significant differences, in terms of antimicrobial sensitivity, depending on the classes of antibiotics used. Of the three strains belonging to the genus *Enterobacter*, two were resistant to the action of four beta-lactams (ampicillin, amoxicillin/clavulanic acid, cephalexin, cephalothin), and one was resistant to three beta-lactams (amoxicillin/clavulanic acid, cephalexin, cephalothin). To aminoglycosides, quinolones, furans, and tetracyclines, all strains were sensitive.

*Providencia rettgeri* was resistant to four beta-lactams (ampicillin, amoxicillin/clavulanic acid, cephalexin, cephalothin), an aminoglycoside (flumequine), and tetracycline (35.3%), and to the other 11 molecules (64.5%) it was sensitive. *Pseudomonas oleovorans* was resistant only to four beta-lactams (ampicillin, amoxicillin/clavulanic acid, cephalexin, cephalothin), being sensitive to the other 13 antibiotics (76.5%) (Table 2).

#### 2.2.2. Chișinău Criș–Sălișteanca Hunting Ground

Analyzing the results obtained, following the performance of laboratory tests, with the strains taken from the animals in the Chișinău Criș-Sălișteanca area, extremely significant differences were found, compared to the results obtained with the strains from the animals in the Socodor area. Additionally, significant differences were found between the strains studied, within the same species, but also depending on the 17 antibiotics tested. The experiments performed showed that for 14 of the 17 tested compounds (82.3%), namely ampicillin, amoxicillin/clavulanic acid, cefquinome, cefoperazone, ceftiofur, imipenem, gentamicin, amikacin, neomycin, enrofloxacin, marbofloxacin, tetracycline, nitrofurantoin, trimethoprim/sulfamethoxazole, the sensitivity was 100%.

The only antibiotic to which the resistance was 100% was cephalexin, while resistence to cephalothin was present in eight of the 17 strains tested (47.1%), the other nine being moderately sensitive. Among the aminoglycosides, two strains of *Escherichia coli* (11.8%) and one strain of *Salmonella* spp. were resistant to flumequine (5.9%) (Table 2).

Although potentially pathogenic germs were isolated, most of the results, in this case, indicated that cervid species do not constitute an important infectious carrier for the antibiotic-resistant microorganisms.

#### 2.2.3. Nadăș Hunting Ground

Results similar to those recorded in the samples taken from the animals in the Chișinău Criș-Sălișteanca area were also found following the experiments on strains isolated and identified from the samples collected from deer in the Nadăș hunting ground. The experiments were performed on 15 bacterial strains, belonging to some Gram-negative species, included in the genera mentioned above, namely *Escherichia* (10), *Salmonella* (3), and *Enterobacter* (2) (Table 2).

Analyzing the obtained results, it is found that the ten strains of *Escherichia coli* were, in general, sensitive to most antimicrobials, with very few exceptions. Of the classes of antimicrobials tested, all but one strain were resistant to one, two, and up to four beta-lactams. Thus, eight strains (80%) were resistant to cephalexin; six (60%) were resistant to cephalothin; one strain was moderately sensitive; four strains (40%) were resistant to cefquinome; three were resistant to cefoperazone; two were resistant to ampicillin (20%); and only one was resistant to amoxicillin/clavulanic acid. Among the other classes of substances, resistance to aminoglycosides (flumequine) was also found in two strains and to a single quinolone (enrofloxacin) (Table 2).

The three strains of *Salmonella* spp. were resistant only to beta-lactams, respectively, to cephalexin in all three strains, and to cephalothin in only one strain; the other two showed moderate resistance. The results were also similar for *Enterobacter* spp.; the two strains studied showed resistance only to cephalexin.

By category, analyzing the results obtained in this study, it appears that the most effective antibiotics were amikacin and imipenem among the beta-lactams, to which all 47 bacterial strains taken in the study were sensitive (100%), followed by nitrofurantoin, to which 45 strains (95.7%) were sensitive, neomycin, to which 44 strains (93.6%) were sensitive, ceftiofur, to which 43 strains (91.5%) were sensitive, and trimethoprim/sulfamethoxazole and marbofloxacin, to which 42 strains were sensitive. The results were also different from those obtained (89.4%).

The most common antibiotic resistence was towards cephalexin, as 45 of the 47 strains (95.7%) belonging to all the species studied were resistant, and only two were sensitive (4.3%). A total of 27 strains were resistant to cephalothin (57.5%); 17 showed moderate resistance (36.2%); and only two were sensitive (4.3%). Significant resistance was also found to ampicillin in 26 of the strains studied (55.3%); the other 21 were sensitive (44.7%).

Fluoroquinolones are considered broad-spectrum antibiotics against both Gram-positive and Gram-negative bacteria. To enrofloxacin, all strains of bacteria isolated, belonging to the species *Salmonella* spp., *Enterobacter* spp., *Providencia rettgeri*, and *Pseudomonas oleovorans*, were sensitive (100%). Five strains of *Escherichia coli*, isolated from animals in the Socodor area and one from Nadăș (12.8%), were resistant. Only five strains (10.6%) were resistant to marbofloxacin, out of the ten isolated from the animals in the Socodor area; the other 42 (89.4%) were sensitive, regardless of where the samples came from or the bacterial species.

### 2.3. Antimicrobial Susceptibility Results of Some Gram-Positive Bacterial Species with the Vitek 2 Compact

Results regarding the antimicrobial susceptibility of some Gram-positive bacterial strains were obtained after performing experiments on a number of 45 bacterial species, classified into four genera, namely *Staphylococcus*, *Aerococcus*, *Enterococcus*, and *Kocuria*.

From the genus *Staphylococcus*, 37 strains were studied, belonging to the following five species: *S. aureus* (one strain), *S. lentus* (10 strains), *S. sciuri* (11 strains), *S. vitulinus* (seven strains), and *S. xylosus* (eight strains). These five species, except the *S. aureus* species, isolated from a single animal from the Chișinău Criș–Sălișteanca hunting grounds, were isolated from samples collected from deer and fawns from all three hunting grounds (Socodor, Chișinău Criș–Sălițeanca, and Nadăș).

From the genus *Aerococcus*, four strains were tested, belonging to the species *A. viridans*, isolated from samples collected from animals in two of the hunting grounds (Chișineu Criș Sălișteaanca and Socodor). From the genus *Enterococcus*, a single strain, belonging to the species *E. faecium*, was isolated from a deer shot in Socodor, and from the genus *Kocuria*, three strains were isolated from animals in the Chișinău Criș–Sălișteanca and Socodor areas.

#### 2.3.1. Socodor Hunting Ground

After carrying out the susceptibility tests on the samples taken from the Socodor area, it was found that the results are quite similar to those obtained with Gram-negative strains. They had a variable susceptibility from one strain to another and from one species to another (Table 3).

**Figure 2:**
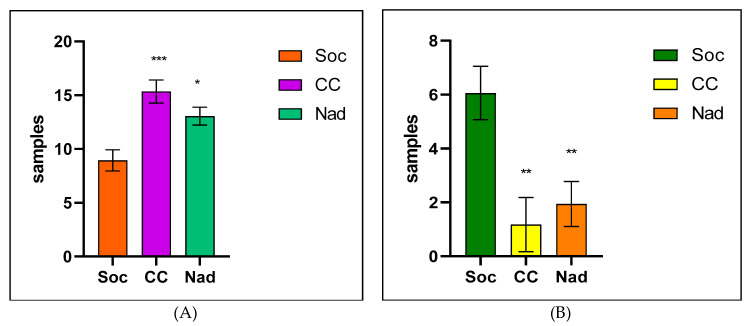
Comparative Sensitivity (**A**)/Resistance (**B**), in studied hunting grounds after the Vitek-2 Compact method (Where: *** means *p* < 0.001, ** *p* < 0.01 and * *p* < 0.05; Soc = Socodor, CC = Chișineu–Criș, Nad = Nadăs hunting grounds).

The statistical analysis of sensitivity vs. resistance in the case of the Vitek-2 Compact (BioMérieux, France) method reconfirmed the presence of higher resistance to antimicrobials in the Socodor hunting ground (** *p* < 0.01), compared with the other two studied areas (Figure 2).

After analyzing the results, of all the species isolated and identified as belonging to the genus *Staphylococcus*, only two strains of *S. lentus* were resistant to one (enrofloxacin), respectively, two aminoglycosides (gentamicin and enrofloxacin), and three other strains showing intermediate resistance.

The results were also different from those obtained following the research carried out on Gram-negative strains, isolated from the same hunting ground, in which five of the *E. coli* strains isolated were resistant to two or even three of the four tested aminoglycosides. Resistance to erythromycin, tilmicosin, tylosin, and clindamycin (macrolide class), of the two strains of *Aerococcus viridans*, was 100%. A 100% resistance was also found in one of the strains of *Kocuria kristinae*, while in the second strain, resistance was present to three of the four macrolides (75%); therefore, resistance to erythromycin was found to be moderate.

Additionally, of the ten strains of staphylococci studied, in three strains, one of *S. lentus* and two of *S. xylosus*, resistance to macrolides was 100%. Another strain of *S. lentus* was resistant to tilmicosin, tylosin, and clindamycin and showed moderate resistance to erythromycin. The only species sensitive to all macrolides tested was *S. Sciuri*, and one of the strains of *S. vitulinus* showed a resistance of 75%, and for the other two 25%, respectively, they were resistant to a single substance (25%) and sensitive in the other three (75%).

A proportion of 66.7% of the 15 bacterial strains tested showed resistance to tetracycline. The resistance was 100% in *E. faecium*, in the two strains of *A. viridans*, and in the two strains of *Kocuria kristinae*.

Trimethoprim/Sulfamethoxazole can be a therapeutic option in the case of multiresistant *S. aureus* strains, taking into account the high sensitivity to in vitro testing. In this study, in deer and fawns from the Socodor area, since only species with minor implications in veterinary pathology (wild phenotype) were isolated, only one strain of *S. lentus* was resistant to trimethoprim/sulfamethoxazole; the others were all sensitive (Table 3).

#### 2.3.2. Chișinău Criș–Sălișteanca Hunting Ground

The studies carried out on the samples collected from the Chișinău Criș–Sălișteanca hunting grounds did not reveal a significant susceptibility in the species studied, regardless of the class of antimicrobials tested.

For aminoglycosides, the results were similar to those found in the samples taken from the Socodor area. Thus, with the exception of one strain of *S. sciuri*, which showed intermediate resistance to two aminoglycosides (amikacin and kanamycin), all other strains, regardless of species, were 100% sensitive.

Against fluoroquinolones (enrofloxacin), the presence of resistant strains was limited to one strain of *Kocuria*/*Dermacoccus* spp. and one strain of *S. Lentus*. Another strain of this species showed intermediate resistance.

All other strains, regardless of species, were susceptible. Instead, most strains were resistant to erythromycin. Additionally, the *S. aureus* strain was resistant to three of the four macrolides (tylosin, tilmicosin, and clindamycin); *A. viridans* was resistant to erythromycin; and the other staphylococcal strains were resistant to one, two, or even three macrolides. However, three strains were sensitive to all macrolides tested.

The acquired data demonstrates that in addition to the diversity of *Staphylococcus* strains isolated, mostly non-pathogenic, regardless of whether they were collected from the nasal cavity or the rectum, there was a wide variability of resistance phenotypes to various classes of antibiotics. Among the other antibiotics, the resistance to tetracycline was also found in three strains, namely one strain of *A. viridans*, one of *Kocuria*, and one of *S. xylosus*. Only one strain of *S. xylosus* was resistant to florfenicol, and all strains were sensitive to sulfonamides (trimethoprim/sulfamethoxazole), (Table 3).

#### 2.3.3. Nadăș Hunting Ground

In the strains collected from the animals from the Nadăș hunting grounds, all belonging to the genus *Staphylococcus*, the sensitivity was 100% to the four aminoglycosides and 100% to enrofloxacin and trimethoprim/sulfamethoxazole. Only seven strains were resistant to one (five strains) or two macrolides, namely tilmicosin and tylosin, and a strain of *S. lentus* was resistant to tetracycline and florfenicol (Table 3).

By substance category, the most effective antibiotics were the following: amikacin and imipenem, to which all 47 strains studied (100%) were sensitive, followed by nitrofurantoin, to which 45 were sensitive (95.7%), neomycin, to which 44 strains (93.6%) were sensitive, ceftiofur, to which 43 strains (91.5%) were sensitive, and trimethoprim/sulfamethoxazole and marbofloxacin, to which 42 strains (89.4%) were sensitive.

## 3. Discussion

*Escherichia coli* was selected for testing due to its ability to easily acquire and transfer antibiotic resistance genes but also because *E. coli* is a commensal bacterium, present in the normal intestinal flora of domestic and wild animals, and was also frequently isolated and identified in the samples collected in this research [18,20].

Similar results to those obtained in the Socodor area were also obtained in research carried out in Portugal, on 72 samples of feces, collected from different species of wild animals. In the research of the 72 samples, *E. coli* was isolated and identified in 56 samples (78%); the authors concluded that the intestinal tract of wild animals is a reservoir of antibiotic resistance genes, especially for ampicillin, tetracycline, streptomycin, and trimethoprim/sulfamethoxazole [21].

*Escherichia coli* was also isolated from the feces of wild and domestic animal species, in South Africa. Subsequently, the isolated strains were tested for antibiotic resistance, using the Kirby–Bauer disk diffusimetric method, against chloramphenicol, nalidixic acid, ampicillin, streptomycin, sulfafurazole, and tetracycline. No significant differences in the antibiotic resistance patterns between the strains isolated from wild animals and domestic animals, which grazed together, were found in said study [22,23].

The results of this study suggest that there may be an exchange of antibiotic-resistant bacteria and resistance genes between wild and domestic animals that graze together. This is confirmed by the fact that in wild animal communities, where domestic animals are also present, including a human presence, despite the perception of a low risk of development of resistance to antimicrobials, resistant pathogens may still emerge; these animals are becoming a growing public health concern [22,24].

In a study on the resistance of *Enterobacter* spp., authors found that the species belonging to this genus are resistant to ampicillin, amoxicillin, amoxicillin/clavulanic acid, first-generation cephalosporins, and cefoxitin, due to the production of AmpC beta-lactamases. The authors stated that most *Enterobacter* spp. isolates were sensitive to fluoroquinolones, trimethoprim/sulfamethoxazole, aminoglycosides, and carbapenems, and because of the high risk of developing resistance during treatment, all severe infections should be carefully monitored during therapy [25].

The results obtained, especially in species belonging to the *Enterobacteriaceae* family, are also confirmed by other researchers [7]. According to the literature data, in wild animals, *E. coli* has been most frequently isolated from red deer, roe deer, wild boar, and fallow deer, species considered important carriers of food-borne pathogens that can cause serious illness in humans and contaminate fresh food products. In a study presented in 2020, in Poland, the presence of STEC (Shiga toxin) strains in fallow deer populations was tracked. Thus, during two hunting seasons, autumn–winter of 2017–2018 and 2018–2019, a total number of 94 rectal swabs were taken from the population of fallow deer (*Dama dama*). Following the laboratory examinations, from the samples collected from the studied population (94 deer belonging to the *Dama dama* species), 63 STEC strains were isolated and identified, of which the stx1 and stx2 resistance genes were identified in 21 [26,27].

Monitoring the prevalence of resistance of potentially pathogenic bacteria such as *Escherichia coli* and *Salmonella* spp. in wildlife makes it possible to demonstrate that wildlife has the potential to serve as a source of germs, including bacteria, in which antimicrobial resistance is present. Additionally, a large number of researchers are addressing the issue of the proliferation of antimicrobial-resistant microorganisms in the wild environment and the potential impact on human health and the environment [23,28,29,30,31].

A resistance of 70% was found to ampicillin, cefquinome, and cefoperazone; six strains were resistant (60%) to amoxicillin/clavulanic acid; five strains were resistant (50%); and four strains were resistant to ceftiofur (40%) (see Table 1). Bacteria develop resistance to beta-lactams through a variety of mechanisms. The most common mechanism is represented by the destruction of the drug by beta-lactamase. These enzymes have a higher affinity for the antibiotic than the antibiotic for the target. A second mechanism of bacterial resistance to beta-lactam antibiotics is the modification of the antibiotic’s targets of action, and therefore the targets have a much-reduced affinity for the drug. The last mechanism of resistance is represented by the modification by bacteria of their external membrane so that it loses its permeability to the antibiotic [32].

One strategy to prevent beta-lactamase-mediated resistance is to combine the susceptible beta-lactam with an inhibitor that binds avidly to the inactivating enzyme, preventing its attack on the antibiotic (e.g., clavulanic acid and sulbactam). In a meta-study on the membership of bacteria isolated from the feces of red deer (*Cervus elaphus*), in Poland, the authors identified 458 microorganisms, of which 13 (2.8%) were identified as EHEC strains (*Enterohemorrhagic Escherichia coli*). Of these, no strain was identified as having ESBL (Extended-Spectrum Beta-Lactamase) resistance [33].

The four strains, in which 87.5% resistance was found to seven of the eight beta-lactams, were also resistant to three aminoglycosides (gentamicin, neomycin, and flumequine), to the two quinolones (enrofloxacin, and marbofloxacin), but also to tetracycline and trimethoprim/sulfamethoxazole. Aminoglycosides can play an extremely important role if *Enterobacterales* (*Enterobacterales* carbapenem-resistant germs—CRE) develop resistance to carbapenems. Moreover, it is widely accepted that carbapenem-resistant (CRE) germs are difficult to treat because they do not respond to commonly used antibiotics. Furthermore, CRE is occasionally resistant to all available antimicrobials, constituting a real threat to public health. Consequently, the therapeutic role of conventional aminoglycosides such as gentamicin and amikacin should be reevaluated [34,35,36].

Additionally, numerous studies carried out in various areas of the world confirm the fact that multiple resistance to antimicrobial compounds, within *Escherichia coli* serotypes, can present significant variations, from one geographical area to another and from one isolate to another; the phenomenon is also dependent on the way antimicrobials are used, both in veterinary and human medicine [24].

A study, carried out in 15 areas of the USA on 95 samples collected from piglets, showed, in all strains of *Escherichia coli* isolated and tested against several, antibiotics (clindamycin, penicillin, tiamulin, tilmicosin) increased resistance to oxytetracycline (91.6%), chlortetracycline (78.9%), ampicillin (75.8%), and sulfadimethoxine (68.4%) [37]. Relatively high resistance to ceftiofur was also found in 28.4% of the total isolated strains, as opposed to only 40% in the current study, and to enrofloxacin (35.8%)., In the current experiments, the resistance to enrofloxacin was significantly higher, i.e., 50%.

The noteworthy differences, regarding the antibiotic resistance found between the strains isolated from the samples collected from the animals in the Socodor area and the samples from the animals from the other two hunting grounds, are probably a consequence of human influence, favored by the existence of the Crișul Alb watercourse, a known area, frequented by a large number of people, including fishermen and people coming for relaxation and recreation near the river. Comparatively, analyzing the antimicrobial resistance present in the bacterial strains isolated from male and female fallow deer, from the three hunting grounds taken in the study, it was found that most of them are sensitive, in a large proportion, to all the antimicrobials tested. Additionally, the interface between domestic and wild animals, found within this hunting area, has an important contribution to the emergence of pathogenic bacterial strains.

Analyzing and comparing bacteria from the samples taken in the hunting grounds, in the period 2019–2021, nine strains of *Salmonella* spp. were isolated and identified. Along with *Salmonella* spp., 12 strains of *Escherichia coli* and two strains of *Enterobacter* spp. were also isolated. Results similar to these findings were also reported, following a study on 117 wild animals (63 canids, 25 mustelids, 24 birds, and five ungulates). A variety of species including strains of *Salmonella* spp. (4.3%) were identified, of which the *S. typhimurium* serovar was isolated most frequently. Following that determination of susceptibility to antimicrobial compounds, it was found that all 88 strains studied presented resistance/intermediate resistance to at least one class of antibiotics, and the highest values of resistance were observed for the tetracycline class, with the authors confirming that resistant serotypes are also present in wild animals. [15,38]. Additionally, a different study included in the National Cervical Health Surveillance Program in Norway obtained results close to those mentioned above [39].

Analyzing the results obtained with the Vitek-2 equipment, on the strains isolated and identified from deers *Dama dama* from the three hunting funds, a significant difference was found between the isolates, which confirms the results obtained by the disc-diffusimetric method. Thus, in *Escherichia coli* strains, isolated from samples taken from animals in the Socodor area, resistance was present in all strains and in all classes of substances, compared to the samples obtained from animals in the other areas, where the phenomenon was significantly lower, most of the strains being sensitive to the tested substances.

The results of this study were also confirmed in a different study on the antimicrobial resistance of *Escherichia coli*, in strains isolated from deer feces. The authors tested the phenotypic resistance of the *Escherichia coli* species, isolated from fecal samples collected from 879 white-tailed deer (*Odocoileus virginianus*), over a period of ten years. The results obtained demonstrated an increase in the prevalence of multiresistant *E. coli*, during the study period, from 0% to 2.2% and 3.7%, during the years 2006, 2012, and 2016, respectively, including broad-spectrum cephalosporins and fluoroquinolones, which are results significantly influenced by the habitat of the deer, which was specifically mentioned to contain contaminated surface water [13]. Against fluoroquinolones, respectively, enrofloxacin, 20% of the 15 isolated strains exhibited resistance; 40% revealed an intermediate (moderate) resistance; and the remaining 40% were sensitive. Most of the time, the mechanism consisted of the development of one or more mutations in the target DNA gyrase, so that the antibacterial agent no longer intervened in the activity of this enzyme [32].

In staphylococci, out of the ten strains, five were resistant, and five were sensitive. Among the sensitive ones, the species *S. sciuri*, two strains of *S. vitulinus*, and two of *S. xylosus* were included. The sensitivity of *Staphylococcus* strains to florfenicol was 100%, an aspect also observed in the two strains of *A. viridans*, as well as in *E. faecium*. The only strains which presented resistance (100%) belonged to the species *Kocuria kristinae*. Bacteria become resistant to florfenicol by developing mutations in RNA polymerase, resulting in an enzyme unable to bind to the antibiotic [3].

In recent years, resistance to cephalosporins, reported in species belonging to the *Enterobacteriaceae* family, has increased significantly, precisely due to the spread of beta-lactamases (β-lactamases) with extended-spectrum (ESBL). The main types of beta-lactamases, whose presence has been reported in *Enterobacteriaceae*, include AmpC beta-lactamases, extended-spectrum beta-lactamases (ESBLs), and carbapenemases. AmpC beta-lactamases confer resistance to most cephalosporins and monobactams [40].

In a critical review, authors ascertained essential associations relating to the occurrence of antibiotic-resistant bacteria in wildlife and anthropogenic responses into the environment, confirming the present study results. For example, authors confirmed the premise that human-generated inputs into the environment induced the antibiotic resistance occurrence in bacteria hosted by free-ranging wildlife [41].

In two noteworthy studies conducted recently on pathotypes and the antimicrobial susceptibility of *Escherichia coli* from wild boars in Tuscany, comparable to the described findings, the authors indicated that wild mammals might act as reservoirs of resistance and virulence, which could be easily transferred between different ecosystems. This assertion was confirmed by genetic studies conducted where it was observed with certainty that wild boars could transfer antimicrobial-resistant *E. coli* to domestic animals and humans [42,43].

As other authors considered, wild deer could serve as a sentinel species for the surveillance of AMR in the Romanian ecosystems. The deer resistance data obtained recently in Scotland revealed that AMR *E. coli* can appear in deer populations not directly exposed to the selective pressure exerted by antibiotherapy. Exactly like in our case, researchers concluded that resistance to critically important antimicrobials was low in the studied deer population, evoking no immediate peril regarding human health [44].

## 4. Materials and Method

### 4.1. Samples Source

The research was carried out during the years 2017–2021, during open hunting season, in the autumn–winter (the months of October, November, December, January, and February), the samples being taken from males (bucks) and females (deer) of fallow deer (*Dama dama*).

The samples were collected from individuals of different ages, immediately after shooting, from three hunting grounds from the Western part of Romania, two hunting grounds from Arad County, Socodor (7601 hectares) and Chișinău-Criș–Sălișteanca (6008 ha), and one from Timiș County, Nadăș (9953 ha). This area is considered one the largest populations of fallow deer on the continent, considered “the fallow deer paradise in Europe”.

Samples were collected from a total of 120 animals. The samples were collected from each deer or fawn, 240 fresh samples in total, from the nasal cavity and the rectum of the animals and directed to the microbiology lab to follow diffusivity (by the Kirby–Bauer disc-diffusimetric method) and the antimicrobial susceptibility of bacterial strains isolated (by Vitek-2 Compact).

### 4.2. The Antimicrobial Susceptibility of Bacterial Strains, Isolated by the Diffusimetric Method

The Kirby–Bauer disc-diffusimetric method was used according to CLSI (2018) guidelines and Annex, using fresh 18–24 h cultures on nutrient agar. For the result accuracy, a positive control strain *E. coli* ATCC 25922 (Thermo Fisher Scientific, Lake Charles, LA, USA) and a negative control were used [45,46,47].

In this case, only Gram-negative species were studied for susceptibility testing. Table 4 presents the bacterial species against which the antimicrobial sensitivity was tested by the diffusimetric method.

All bacterial strains studied were tested for sensitivity to the following antimicrobial substances: beta-lactams (ampicillin, amoxicillin + clavulanic acid, cefuroxime, and ceftazidime), fluoroquinolones (ciprofloxacin), aminoglycosides (gentamicin), and furans (nitrofurantoin and trimethoprim/sulfamethoxazole). From the stock cultures, belonging to the genera *Escherichia*, *Enterobacter*, *Salmonella*, *Pseudomonas*, and *Providencia*, kept at −50 °C in brain heart broth (BHI Oxoid broth) and glycerol, before each experiment, seeds were made on Müeller–Hinton agar in Petri dishes; then, the plates were incubated at 37 °C for 24 h. Next, the bacterial suspension was prepared, which was brought to the standard turbidity of 0.5 Mc Farland, after which the Petri dishes were seeded with Müeller–Hinton agar.

After the moment of calibration, the plates were seeded, for a maximum of 15 min, by flooding the surface of the solid medium. After the absorption of the inoculum (approximately five minutes), the 25–35 μg micro tablets (Oxoid, Hampshire, UK) with the antimicrobial substance were deposited, using the dispenser; then, the plates were incubated at 37 °C for 24 h. Afterward, the halo diameter around the micro tablets with the antimicrobial compound was measured, and the categories of sensitivity were established for each individual strain, as follows: diameter of the halo below 1 mm—resistant strain, between 2 and 5 mm—moderately sensitive stem, and over 6 mm—sensitive stem. For testing control, reference microbial strains, with unaltered natural sensitivity to antibiotics, were used.

### 4.3. The Antimicrobial Susceptibility of the Isolated Bacterial Strains by Vitek-2 Compact (BioMérieux, Craponne, France)

The study of the susceptibility to the action of the main classes of antimicrobial substances was carried out on Gram-negative (the AST codes of the Vitek2 cards: AST-GN96 and GN97) and Gram-positive species (the AST code: GP79) isolated from deer and fawns. To carry out the determinations, from a total of 240 strains isolated from 120 animals, 47 Gram-negative strains and 45 Gram-positive strains were studied. Table 5 presents Gram-negative and Gram-positive species tested for antimicrobial susceptibility.

For the Gram-negative isolates, the sensitivity to 17 antimicrobial substances, existing in the Vitek-2 Compact equipment cards, was tested as follows: Beta-lactams: AMP—Ampicillin, AMC—Amoxicillin/Clavulanic acid, CN—Cefalexin, CF—Cephalothin, CEC—Cefquinone, CFP—Cefoperazone, FUR—Ceftiofur, IPM—Imipenem; Aminoglycosides: GM—Gentamicin, AK—Amikacin, N—Neomycin, UMB—Flumequine; Quinolones: ENR—Enrofloxacin, MAR—Marbofloxacin; Tetracycline: TE—Tetracycline; Furans: FT—Nitrofurantoin; Sulfonamides: SXT—Trimethoprim/Sulfamethoxazole (Supplementary Table S1).

For Gram-positive bacteria, the card included 17 substances, of which 12 reacted, as follows: Aminoglycosides: AN—Amikacin, GM—Gentamicin, K—Kanamycin, N—Neomycin; Quinolones: ENR—Enrofloxacin; Macrolides: E—Erythromycin, TIL—Tilmicosin, TYL—Tylosin, CM—Clindamycin; Tetracycline: TE—Tetracycline; Fenicols: FLO—Florfenicol; Sulfonamides: SXT—Trimethoprim/Sulfamethoxazole (Supplementary Table S1).

### 4.4. The Statistical Analysis

All the values were analyzed statistically by one-way ANOVA with Bonferroni post-test using GraphPad Prism 9.1 for Windows (GraphPad Software, San Diego, CA, USA). Results are expressed as mean + SEM; all values lower than *p <* 0.05 are considered statistically significant.

## 5. Conclusions

Compared to the strains isolated from the hunting grounds Chișinău Criș–Sălițeanca and Nadăș, *Escherichia coli*, isolated from the animals in the Socodor hunting grounds, showed higher statistically significant resistance to most of the antimicrobial molecules tested, composed within 50 and 90% (*p* < 0.001).

Monitoring the prevalence of resistance in some potentially pathogenic bacteria, such as *Escherichia coli* and *Salmonella* spp., present in wildlife in certain sylvatic areas demonstrates that wildlife has the potential to serve as a source of germs, including bacteria in which antimicrobial resistance is present. Although studies indicate that some *Enterobacter* species are resistant to β-lactam antibiotics due to the production of AmpC beta-lactamases, the results obtained do not confirm these claims, possibly because the wild *Enterobacter* phenotype generally circulates in the fallow deer population.

## Figures and Tables

**Table 1:** Antimicrobial susceptibility of species isolated from the studied three hunting grounds.

Antimicrobial Susceptibility of Species Isolated from the Socodor Hunting Ground										
Antimicrobial	Bacterial Species/Susceptibility/Drug									
	*E. coli*					*P. oleovorans*	*P. rettgeri*	*Enterob.* spp.	*Enterob. aerogenes*	*Enterob.* spp.
	1	2	3	4	5					
Ampicillin	R	R	R	R	R	S	S	R	R	S
Amoxi/Clavulanic ac	R	R	R	R	R	S	S	R	R	S
Cefuroxime	R	R	S	R	R	S	R	S	S	S
Ceftazidim	S	S	S	S	S	S	S	R	S	S
Ciprofloxacin	R	R	R	R	R	S	S	S	S	R
Gentamicin	R	R	S	R	R	S	S	S	S	S
Nitrofurantoin	S	S	R	S	R	S	S	R	S	S
Trimetho/Sulfametho	S	R	S	R	R	S	S	S	S	S
Chloramphenicol	S	R	S	R	R	S	R	S	R	S
Azithromycin	R	R	R	R	R	S	S	R	R	S
**Resistant** (%)	**60**	**80**	**50**	**80**	**90**	**0**	**20**	**50**	**40**	**10**
**Sensitive** (%)	**40**	**20**	**50**	**20**	**10**	**100**	**80**	**50**	**60**	**90**
**Antimicrobial Susceptibility of Species Isolated from the Chișinău Criș–Sălișteanca Hunting Ground**										
**Antimicrobial**	***E. coli***					***Salmonella* spp.**			***Enterobacter* spp.**	
	**1**	**2**	**3**	**4**	**5**	**1**	**2**	**3**	**1**	**2**
Ampicillin	S	**R**	**R**	S	S	S	S	S	S	S
Amoxi/Clavulanic ac	S	S	S	S	S	S	S	S	S	S
Cefuroxime	S	S	S	S	S	S	S	S	S	S
Ceftazidim	S	S	S	S	S	S	S	S	S	S
Ciprofloxacin	S	S	S	S	S	S	S	S	S	S
Gentamicin	S	S	S	S	S	S	S	S	S	S
Nitrofurantoin	S	S	S	S	S	S	S	S	S	S
Trimetho/Sulfametho	S	S	S	S	S	S	S	S	S	S
Chloramphenicol	S	S	S	S	S	S	S	S	S	S
Azithromycin	S	S	S	S	S	S	S	S	S	S
**Resistant** (%)	**0**	**10**	**10**	**0**	**0**	**0**	**0**	**0**	**0**	**0**
**Sensitive** (%)	**100**	**90**	**90**	**100**	**100**	**100**	**100**	**100**	**100**	**100**
**Antimicrobial Susceptibility of Species Isolated from the Nadăș Hunting Ground**										
**Antimicrobial**	***E. coli***					***Salmonella* spp.**			***Enterobacter* spp.**	
	**1**	**2**	**3**	**4**	**5**	**1**	**2**	**3**	**1**	**2**
Ampicillin	**S**	**R**	**R**	**S**	**S**	**S**	**S**	**R**	**S**	**S**
Amoxi/Clavulanic ac	**S**	**S**	**S**	**S**	**S**	**S**	**S**	**S**	**S**	**S**
Cefuroxime	**S**	**S**	**R**	**R**	**S**	**S**	**S**	**S**	**R**	**R**
Ceftazidim	**S**	**S**	**R**	**R**	**S**	**S**	**S**	**S**	**S**	**S**
Ciprofloxacin	**S**	**S**	**S**	**S**	**S**	**S**	**S**	**S**	**S**	**S**
Gentamicin	**S**	**R**	**S**	**S**	**S**	**S**	**R**	**R**	**S**	**S**
Nitrofurantoin	**S**	**S**	**S**	**S**	**S**	**S**	**S**	**S**	**S**	**S**
Trimetho/Sulfametho	**S**	**S**	**S**	**R**	**S**	**S**	**S**	**S**	**S**	**S**
Chloramphenicol	**S**	**S**	**S**	**S**	**S**	**S**	**S**	**S**	**S**	**S**
Azithromycin	**S**	**S**	**S**	**S**	**R**	**S**	**R**	**S**	**R**	**R**
**Resistant** (%)	**0**	**20**	**30**	**30**	**10**	**0**	**20**	**20**	**20**	**20**
**Sensitive** (%)	**100**	**80**	**70**	**70**	**90**	**100**	**80**	**80**	**80**	**80**

Legend: Red: R—resistant; Green: S—sensitive.

**Table 2:** Antimicrobial susceptibility of Gram-negative species isolated from the three studied hunting grounds.

Antimicrobial Susceptibility of Species Isolated from the Socodor Hunting Ground																	
Species	Antimicrobial Class																
	Beta-Lactamins								Aminoglicosides				Quinolones		Tcy.	Fourans	
	AMP	AMC	CN	CF	CEC	CFP	FUR	IPM	GM	AK	N	UMB	ENR	MAR	TE	FT	SXT *
*Ps. oleovorans*	**R**	**R**	**R**	**R**	**S**	**S**	**S**	**S**	**S**	**S**	**S**	**S**	**S**	**S**	**S**	**S**	**S**
*Pr. rettgeri*	**R**	**R**	**R**	**R**	**S**	**S**	**S**	**S**	**S**	**S**	**S**	**R**	**S**	**S**	**R**	**S**	**S**
*Enterobacter* sp.	**R**	**R**	**R**	**R**	**S**	**S**	**S**	**S**	**S**	**S**	**S**	**S**	**S**	**S**	**S**	**S**	**S**
*Enterobacter* sp.	**S**	**R**	**R**	**R**	**S**	**S**	**S**	**S**	**S**	**S**	**S**	**S**	**S**	**S**	**S**	**S**	**S**
*Ent. aerogenes*	**R**	**R**	**R**	**R**	**S**	**S**	**S**	**S**	**S**	**S**	**S**	**S**	**S**	**S**	**S**	**S**	**S**
*Escherichia coli*	**S**	**S**	**R**	**I**	**R**	**S**	**S**	**S**	**S**	**S**	**S**	**S**	**S**	**S**	**S**	**S**	**S**
*Escherichia coli*	**R**	**S**	**R**	**I**	**R**	**R**	**S**	**S**	**S**	**S**	**S**	**S**	**S**	**S**	**R**	**S**	**S**
*Escherichia coli*	**R**	**R**	**R**	**R**	**R**	**R**	**R**	**S**	**R**	**S**	**R**	**R**	**R**	**R**	**R**	**S**	**R**
*Escherichia coli*	**R**	**R**	**R**	**R**	**R**	**R**	**R**	**S**	**R**	**S**	**R**	**R**	**R**	**R**	**R**	**R**	**R**
*Escherichia coli*	**R**	**R**	**R**	**R**	**R**	**R**	**R**	**S**	**R**	**S**	**S**	**R**	**R**	**R**	**R**	**R**	**R**
*Escherichia coli*	**R**	**R**	**R**	**R**	**R**	**R**	**R**	**S**	**R**	**S**	**R**	**R**	**R**	**R**	**R**	**S**	**R**
*Escherichia coli*	**R**	**R**	**R**	**R**	**S**	**S**	**S**	**S**	**R**	**S**	**S**	**R**	**R**	**R**	**R**	**S**	**R**
*Escherichia coli*	**S**	**S**	**R**	**R**	**S**	**S**	**S**	**S**	**S**	**S**	**S**	**S**	**S**	**S**	**S**	**S**	**S**
*Escherichia coli*	**R**	**S**	**R**	**R**	**S**	**R**	**S**	**S**	**S**	**S**	**S**	**S**	**S**	**S**	**R**	**S**	**S**
*Escherichia coli*	**S**	**S**	**R**	**I**	**S**	**S**	**S**	**S**	**S**	**S**	**S**	**S**	**S**	**S**	**S**	**S**	**S**
**Antimicrobial Susceptibility of Species Isolated from the Chișinău Criș–Sălișteanca hunt Ground**																	
**Species**	**Beta-Lactamins**								**Aminoglicosides**				**Quinolones**		**Tcy.**	**Fourans**	
	**AMP**	**AMC**	**CN**	**CF**	**CEC**	**CFP**	**FUR**	**IPM**	**GM**	**AK**	**N**	**UMB**	**ENR**	**MAR**	**TE**	**FT**	SXT *
*Escherichia coli*	**S**	**S**	**R**	**R**	**S**	**S**	**S**	**S**	**S**	**S**	**S**	**S**	**S**	**S**	**S**	**S**	**S**
*Escherichia coli*	**S**	**S**	**R**	**I**	**S**	**S**	**S**	**S**	**S**	**S**	**S**	**S**	**S**	**S**	**S**	**S**	**S**
*Escherichia coli*	**S**	**S**	**R**	**R**	**S**	**S**	**S**	**S**	**S**	**S**	**S**	**S**	**S**	**S**	**S**	**S**	**S**
*Escherichia coli*	**S**	**S**	**R**	**I**	**S**	**S**	**S**	**S**	**S**	**S**	**S**	**S**	**S**	**S**	**S**	**S**	**S**
*Escherichia coli*	**S**	**S**	**R**	**I**	**S**	**S**	**S**	**S**	**S**	**S**	**S**	**S**	**S**	**S**	**S**	**S**	**S**
*Escherichia coli*	**S**	**S**	**R**	**R**	**S**	**S**	**S**	**S**	**S**	**S**	**S**	**S**	**S**	**S**	**S**	**S**	**S**
*Escherichia coli*	**S**	**S**	**R**	**I**	**S**	**S**	**S**	**S**	**S**	**S**	**S**	**S**	**S**	**S**	**S**	**S**	**S**
*Escherichia coli*	**S**	**S**	**R**	**R**	**S**	**S**	**S**	**S**	**S**	**S**	**S**	**S**	**S**	**S**	**S**	**S**	**S**
*Escherichia coli*	**S**	**S**	**R**	**R**	**S**	**S**	**S**	**S**	**S**	**S**	**S**	**S**	**S**	**S**	**S**	**S**	**S**
*Escherichia coli*	**S**	**S**	**R**	**I**	**S**	**S**	**S**	**S**	**S**	**S**	**S**	**S**	**S**	**S**	**S**	**S**	**S**
*Escherichia coli*	**S**	**S**	**R**	**R**	**S**	**S**	**S**	**S**	**S**	**S**	**S**	**R**	**S**	**S**	**S**	**S**	**S**
*Escherichia coli*	**S**	**S**	**R**	**R**	**S**	**S**	**S**	**S**	**S**	**S**	**S**	**R**	**S**	**S**	**S**	**S**	**S**
*Salmonella* sp.	**S**	**S**	**R**	**I**	**S**	**S**	**S**	**S**	**S**	**S**	**S**	**S**	**S**	**S**	**S**	**S**	**S**
*Salmonella* sp.	**S**	**S**	**R**	**R**	**S**	**S**	**S**	**S**	**S**	**S**	**S**	**R**	**S**	**S**	**S**	**S**	**S**
*Salmonella* sp.	**S**	**S**	**R**	**I**	**S**	**S**	**S**	**S**	**S**	**S**	**S**	**S**	**S**	**S**	**S**	**S**	**S**
*Enterobacter* sp.	**S**	**S**	**R**	**I**	**S**	**S**	**S**	**S**	**S**	**S**	**S**	**S**	**S**	**S**	**S**	**S**	**S**
*Enterobacter* sp.	**S**	**S**	**R**	**I**	**S**	**S**	**S**	**S**	**S**	**S**	**S**	**S**	**S**	**S**	**S**	**S**	**S**
**Antimicrobial Susceptibility of Species Isolated from the Nadăș Hunting Stock**																	
**Species**	**Beta-lactamins**								**Aminoglicosides**				**Quinolones**		**Tcy.**	**Fourans**	
	**AMP**	**AMC**	**CN**	**CF**	**CEC**	**CFP**	**FUR**	**IPM**	**GM**	**AK**	**N**	**UMB**	**ENR**	**MAR**	**TE**	**FT**	SXT *
*Escherichia coli*	**R**	**R**	S	S	S	S	S	S	S	S	S	S	S	S	S	S	S
*Escherichia coli*	S	S	S	S	S	S	S	S	S	S	S	S	S	S	S	S	S
*Escherichia coli*	S	S	**R**	S	S	S	S	S	S	S	S	S	S	S	S	S	S
*Escherichia coli*	S	S	**R**	**R**	**R**	S	S	S	S	S	S	S	S	S	S	S	S
*Escherichia coli*	S	S	**R**	**R**	**R**	**R**	S	S	S	S	S	S	S	S	S	S	S
*Escherichia coli*	S	S	**R**	**I**	**R**	**R**	S	S	S	S	S	S	S	S	S	S	S
*Escherichia coli*	S	S	**R**	**R**	S	S	S	S	S	S	S	**R**	**R**	S	**I**	S	S
*Escherichia coli*	S	S	**R**	**R**	S	S	S	S	S	S	S	S	S	S	S	S	S
*Escherichia coli*	**R**	S	**R**	**R**	S	S	S	S	S	S	S	**R**	S	S	S	S	S
*Escherichia coli*	S	S	**R**	**R**	**R**	**R**	S	S	S	S	S	S	S	S	S	S	S
*Salmonella* sp.	S	S	**R**	**I**	S	S	S	S	S	S	S	S	S	S	S	S	S
*Salmonella* sp.	S	S	**R**	**I**	S	S	S	S	S	S	S	S	S	S	S	S	S
*Salmonella* sp.	S	S	**R**	**R**	S	S	S	S	S	S	S	S	S	S	S	S	S
*Enterobacter* sp.	S	S	**R**	**I**	S	S	S	S	S	S	S	S	S	S	S	S	S
*Enterobacter* sp.	S	S	**R**	**I**	S	S	S	S	S	S	S	S	S	S	S	S	S

Legend: AMP—Ampicillin, AMC—Amoxicillin/Clavulanic Acid, CN—Cephalexin, CF—Cephalothin, CEC—Cefquinone, CFP—Cefoperazone, FUR—Ceftiofur, IPM—Imipenem, GM—Gentamicin, AK—Amikacin, N—Neomycin, UMB—Flumequine, ENR—Enrofloxacin, MAR—Marbofloxacin, TE—Tetracycline, FT—Nitrofurantoin, SXT *—Trimethoprim/Sulfamethoxazole—included in the class of Sulfonamides; Red: R—resistant; Green: S—sensitive; Blue: I—moderately sensitive (intermediate).

**Table 3:** Antimicrobial susceptibility of Gram-positive species isolated from the three studied hunting grounds.

Antimicrobial Susceptibility of Species Isolated from the Socodor Hunting Ground												
Species/Antimicrobial	AN	GM	K	N	ENR	E	TIL	TYL	CM	TE	FLO	SXT
*A. viridans*	S	S	S	S	S	**R**	**R**	**R**	**R**	**R**	S	S
*A. viridans*	S	S	S	S	**I**	**R**	**R**	**R**	**R**	**R**	S	S
*Kocuria kristinae*	S	S	**R**	S	**R**	**R**	**R**	**R**	**R**	**R**	**R**	S
*Kocuria kristinae*	S	S	**R**	S	**I**	**I**	**R**	**R**	**R**	**R**	**R**	S
*E. faecium*	S	S	S	S	**I**	**R**	S	S	**R**	**R**	S	S
*S. lentus*	S	**R**	S	S	**R**	**R**	**R**	S	**R**	**R**	S	**R**
*S. lentus*	S	S	S	S	**R**	**I**	**R**	**R**	**R**	**R**	S	S
*S. lentus*	S	S	S	S	S	**R**	**R**	**R**	**R**	**R**	S	S
*S. lentus*	S	**I**	S	S	**I**	**R**	S	**R**	**R**	**R**	S	S
*S. sciuri*	S	S	S	S	S	S	S	S	S	S	S	S
*S. vitulinus*	S	S	S	S	**I**	S	S	**R**	S	**R**	S	S
*S. vitulinus*	S	S	S	S	**I**	S	**R**	**R**	**R**	S	S	S
*S. vitulinus*	S	S	S	S	S	S	S	S	**R**	S	S	S
*S. xylosus*	S	S	S	S	S	**R**	**R**	**R**	**R**	S	S	S
*S. xylosus*	S	S	S	S	S	**R**	**R**	**R**	**R**	S	S	S
**Antimicrobial Susceptibility of Species Isolated from the Chișinău Criș–Sălișteanca Hunting Ground**												
**Species/Antimicrobial**	**AN**	**GM**	**K**	**N**	**ENR**	**E**	**TIL**	**TYL**	**CM**	**TE**	**FLO**	**SXT**
*A. viridans*	S	S	S	S	S	**R**	S	S	S	S	S	S
*A. viridans*	S	S	S	S	S	S	S	S	S	**R**	S	S
*Kocuria/Dermacoccus* spp.	S	S	S	S	**R**	**I**	S	S	S	**R**	S	S
*S. aureus*	S	S	S	S	S	S	**R**	**R**	**R**	S	S	S
*S. lentus*	S	S	S	S	S	S	S	S	S	S	S	S
*S. lentus*	S	S	S	S	**I**	S	S	S	S	S	S	S
*S. lentus*	S	S	S	S	**R**	S	S	**R**	**R**	S	S	S
*S. sciuri*	S	S	S	S	S	S	**R**	S	**R**	S	S	S
*S. sciuri*	S	S	S	S	S	S	S	**R**	**I**	S	S	S
*S. sciuri*	**I**	S	**I**	S	S	**I**	**R**				S	S
*S. vitulinus*	S	S	S	S	S	S	S	S	S	S	S	S
*S. vitulinus*	S	S	S	S	S	S	S	**R**	**R**	S	S	S
*S. vitulinus*	S	S	S	S	S	S	S	S	**R**	S	S	S
*S. xylosus*	S	S	S	S	S	**R**	S	**R**	S	S	S	S
*S. xylosus*	S	S	S	S	S	**I**	**R**	**R**	**R**	**R**	**R**	S
**Antimicrobial Susceptibility of Gram-Positives Isolated from the Nadăș Hunting Ground**												
**Species/antimicrobial**	**AN**	**GM**	**K**	**N**	**ENR**	**E**	**TIL**	**TYL**	**CM**	**TE**	**FLO**	**SXT**
*A. viridans*	S	S	S	S	**I**	S	**R**	S	S	S	S	S
*A. viridans*	S	S	S	S	S	S	**R**	S	S	S	S	S
*Kocuria kristinae*	S	S	S	S	S	**I**	**R**	**R**	S	**R**	**R**	S
*Kocuria kristinae*	S	S	S	S	S	S	S	**R**	S	S	S	S
*E. faecium*	S	S	S	S	S	S	**R**	**R**	S	S	S	S
*S. lentus*	S	S	S	S	S	S	S	**R**	S	S	S	S
*S. lentus*	S	S	S	S	S	S	S	S	S	S	S	S
*S. lentus*	S	S	S	S	S	S	S	S	S	S	S	S
*S. lentus*	S	S	S	S	S	S	S	S	S	S	S	S
*S. sciuri*	S	S	S	S	S	S	S	S	S	S	S	S
*S. vitulinus*	S	S	S	S	S	S	S	S	S	S	S	S
*S. vitulinus*	S	S	S	S	S	S	S	**R**	S	S	S	S
*S. vitulinus*	S	S	S	S	S	S	S	S	S	S	S	S
*S. xylosus*	S	S	S	S	S	S	S	S	S	S	S	S
*S. xylosus*	S	S	S	S	S	S	S	S	S	S	S	S

Legend: Aminoglycosides (AN—Amikacin, GM—Gentamicin, K—Kanamycin, N—Neomycin); Quinolones (ENR—Enrofloxacin); Macrolides (E—Erythromycin, TIL—Tilmicosin, TYL—Tylosin, CM—Clindamycin); Tetracyclines (TE—Tetracycline); Fenicols (FLO—Florfenicol); Sulfonamides (SXT—Trimethoprim/Sulfamethoxazole); Green: S—sensitive; Red: R—resistant; Blue: I—moderately sensitive (intermediate).

**Table 4:** Bacterial species against which the antimicrobial sensitivity was tested by the diffusimetric method sampled on Müeller–Hinton agar/Sigma-Aldrich (Oxoid, Hampshire, UK).

Crt. No.	Hunting Ground	Bacterial Species	Strains No.	Sampling Place
1.	Socodor	*Escherichia coli*	5	Anal
		*Pseudomonas oleovorans*	1	Nasal
		*Providencia rettgeri*	1	Nasal
		*Enterobacter aerogenes*	1	Nasal
		*Enterobacter* spp.	2	Anal
2.	Chișineu Criș–Sălișteanca	*Escherichia coli*	5	Nasal
		*Salmonella* spp.	3	Nasal
		*Enterobacter* spp.	2	Anal
3.	Nadăș	*Escherichia coli*	5	Nasal
		*Salmonella* spp.	3	Anal
		*Enterobacter* spp.	2	Anal

**Table 5:** Gram-negative and Gram-positive species tested for antimicrobial susceptibility with Vitek-2 Compact Sampled on agar Müeller–Hinton/Sigma-Aldrich (Oxoid, Hampshire, UK).

Crt. No.	Bacterial Species	Strains No.	Sampling Place
Gram-negative			
1	*Escherichia coli*	32	anal
2	*Enterobacter* spp.	6	anal
3	*Enterobacter aerogenes*	1	nasal
4	*Salmonella* spp.	6	nasal
5	*Providencia rettgeri*	1	nasal
6	*Pseudomonas oleovorans*	1	nasal
Total samples		47	
Gram-positives			
1	*Aerococcus viridans*	4	anal
2	*Enterococcus faecium*	1	anal
3	*Kocuria kristinae*	3	nasal
4	*Staphylococcus sciuri*	11	nasal
5	*Staphylococcus lentus*	10	nasal
6	*Staphylococcus vitulinus*	7	nasal
7	*Staphylococcus xylosus*	8	nasal
8	*Staphylococcus aureus*	1	nasal
Total samples		45	

## Data Availability

All data are included in the present manuscript.

## Supplementary Materials

The following supporting information can be downloaded at: https://www.mdpi.com/article/10.3390/antibiotics12040743/s1, Table S1: Antimicrobial substances used for testing Gram-negative and Gram-positive species.
