# Supplementary material for: The Behavior of Some Bacterial Strains Isolated from Fallow Deer Compared to Antimicrobial Substances in Western Romania
Source: Antibiotics (Basel). 2023 Apr 12;12(4):743. doi: 10.3390/antibiotics12040743 (PMC10134966; doi:10.3390/antibiotics12040743)
Supplement: Supplementary file 1 [file antibiotics-12-00743-s001.zip › antibiotics-2335407-supplementary.pdf]

Supplementary Material

Table S1. Antimicrobial substances used for testing Gram-negative and Gram-positive species

Antimicrobial	Code	Concentration (µg/ml)	Limits (≤	≥)	FDA directions for use
**Antimicrobial substances used for testing Gram-negative species**					
Ampicillin	AMP	4, 8, 32	2	32	CSAGNB**
Amoxicillin/Clavulanic acid	AMC	4/2, 16/8, 32/16	2/1	32/16	CSAGNB**
Cefalexin	CN	8, 32, 64	4	64	
Cephalothin	CF	2, 8, 32	2	64	CSAGNB**
Cefquinone	CEQ	0.5, 1.5, 4	0.5	8	N/A**
Cefoperazone	CFP	4, 8, 32	4	64	N/A**
Ceftiofur	CFT	1, 2	1	8	N/A**
Imipenem	IPM	1, 2, 6, 12	0.25	16	*Enterobacter spp., E. coli*
Gentamicin	GM	4, 16, 32	1	16	CSAGNB**
Amikacin	AN	8, 16, 64	2	64	CSAGNB**
Neomycin	N	8, 16, 64	2	64	N/A**
Flumequine	UB	2, 4, 8	1	32	N/A**
Enrofloxacin	ENR	0.25, 1, 4	0.12	4	N/A**
Marbofloxacin	MRB	1, 2	0.5	4	N/A**
Tetracycline	TE	2, 4, 8	1	16	CSAGNB**
Nitrofurantoin	FT	16, 32, 64	16	512	CSAGNB**
Trimetho/ Sulfamethoxaxa	SXT	1/19, 4/76, 16/304	20(1/19)	320(16/304)	*Enterobacter* spp., Eco (+ETEC)**
**Antimicrobial substances used for testing Gram-positive species**					
**Antimicrobial**	**Code**	**Concentration (µg/ml)**	**Limits (≤**	**≥)**	**FDA directions for use**
Amikacin	AN	16, 32, 64	2	64	N/A**
Gentamicin	GM	8, 16, 64	0.5	16	*Staphylococcus* spp.
Kanamycin	K	32, 64, 128	4	64	N/A**
Neomycin	N	16, 32, 64	2	32	N/A**
Enrofloxacin	ENR	1, 2	0.5	4	N/A**
Erythromycin	E	0.25, 0.5, 2	0.25	8	*Staphylococcus* spp.
Tilmicosin	TIL	0.5, 1, 4	0.25	4	N/A**
Tylosin	TI	2, 8, 32	1	32	N/A**
Clindamycin	CM	0.06, 0.25, 1	0.125	4	MSSA**, MSSE**
Tetracycline	TE	0.5, 1, 2	1	16	*Staphylococcus spp.,*
Florfenicol	FFC	2, 4, 16	4	32	N/A**
Trimetho/Sulfamethoxa	SXT	8/152, 16/304, 32/608	10(0.5/9.5)	320(16/304)	N/A**

Legend: FDA = Food and Drug Administration; **CSAGNB = clinical significance aerobic Gram-negative bacilli; **N/A = No specific FDA indications are recommended for use. **Eco (+ETEC) = *E. coli* (including sensitive enterotoxigenic strains involved in traveler's diarrhea); NEG = Negative; POS = Pozitive; **MSSA = Methicillin-susceptible *S. aureus*; **MSSE = Methicillin-susceptible *S. epidermidis*.
